# Deprivation and general practitioners’ working lives: Repeated cross-sectional study

**DOI:** 10.1177/01410768251330076

**Published:** 2025-04-21

**Authors:** Michael Anderson, Jonathan Gibson, Benjamin Walker, Joseph Hutchinson, Katherine Checkland, Matt Sutton

**Affiliations:** 1 Health Organisation, Policy, Economics (HOPE), Centre for Primary Care & Health Services Research, The University of Manchester, Manchester M13 9PL, UK; 2 LSE Health, Department of Health Policy, London School of Economics and Political Science, London WC2A 2AE, UK

**Keywords:** Deprivation, inequalities, job satisfaction, job pressure, workforce planning, workforce satisfaction, primary care, general practice

## Abstract

**Objectives:**

To examine how area deprivation affects the working lives of general practitioners (GPs).

**Design:**

We analysed responses to four repeated cross-sectional surveys between 2015 and 2021. We used linear regression to relate the population deprivation ranking of the GP’s practice to job pressures, job satisfaction, intentions to quit direct patient care and hours worked. We used interval regression to relate reported income from GP work to this same deprivation ranking. We adjusted for GP characteristics, including employment status, gender, age and years qualified.

**Setting:**

Primary medical care in England.

**Participants:**

GPs.

**Main outcome measures:**

Fourteen reported job pressures, 10 domains of job satisfaction, intentions to quit direct patient care, reported income from GP work and hours worked per week.

**Results:**

Deprivation ranking was significantly associated with higher pressures related to perceived problem patients (difference between lowest and highest deprivation = 0.258 on five-point scale, 95% CI: 0.165, 0.350), insufficient resources within the practice (0.229, 95% CI: 0.107, 0.351), and finding a locum (0.260, 95% CI: 0.130, 0.390). Deprivation ranking was also associated with significantly lower reported annual income (−£5,525, 95% CI: −£8,773, −£2,276). There were no statistically significant associations between deprivation ranking and the other outcome measures.

**Conclusions:**

Perceived problem patients, insufficient resources and finding temporary cover are key drivers of GP job pressures in practices serving more deprived populations. GPs in more deprived areas also report lower incomes. These factors should be the target of increased investment and policy interventions to improve recruitment and retention of GPs in these areas.

## Introduction

Deprivation significantly impacts life expectancy, health and wellbeing.^1^ In England, as in many countries, there are substantial disparities in life expectancy across the socioeconomic spectrum. Between 2018 and 2020, the gaps in life expectancy between the least and most deprived areas of England were 9.7 years for males and 7.9 for females.^2^

Although the causes of health inequalities are complex,^3^ a potential significant component is disparity in the supply of high-quality health services. The inverse care law proposes that the availability of quality medical care varies inversely with population need.^4^ A recent analysis of the inverse care law in general practice in England found that practices in the most deprived areas are relatively ‘underfunded, under-doctored, and perform less well on a range of quality indicators compared with practices in wealthier areas’.^5^ Any policy aimed at improving life expectancy in deprived areas and narrowing health inequalities would need to address this inverse care law.

Previous work has shown that greater supply of primary care physicians is associated with improved population health outcomes,^6–8^ and quality of care.^9^ Despite this, areas of greater socioeconomic deprivation in England have fewer general practitioners (GPs) per 1000 population,^10^ higher turnover of GPs^11^ and more problems filling GP vacancies.^12^

Several studies have highlighted difficulties of working in deprived areas. Consultations in deprived areas are often more complex, with some patients perceived as lacking personal, social and material resources to manage their multiple long-term conditions and interventions to support the ‘whole person’ being potentially beneficial.^13^ With more complex patients and fewer staff, GPs working in these environments may be exposed to more stress. This could lead to lower job satisfaction and an increased likelihood of leaving the workforce, further exacerbating the inequalities.

However, there are relatively few studies of how population deprivation affects GPs’ working lives. Experiences from GPs working in severely deprived areas in Scotland reveal that consultations are characterised by greater GP stress when compared with least deprived areas.^14,15^ In a study of GPs in Denmark, burnout was more common among GPs with a higher share of deprived patients on their list.^16^ There is limited quantitative research on the differences in GP experiences based on area deprivation levels and no studies in England. In this study, we use data from multiple large national GP surveys to examine differences in GP working experiences by the deprivation level of the patient list that they serve.

## Methods

### Study cohort

We used data from the eighth (2015), ninth (2017), 10th (2019) and 11th (2021) waves of the GP Worklife Survey (GPWLS). The GPWLS is a national survey of GPs conducted every 2–3 years in England. Details of the survey periods are in Supplementary Table 1.

The eighth, ninth and 10th GPWLSs were conducted by posting the survey to a random sample of GPs (4000 GPs for the eighth and ninth waves, and 4976 GPs for the 10th wave) with an option to conduct an online version. The 11th survey was conducted during the COVID-19 pandemic using an online survey, and respondents were asked to participate in the study through an invitation emailed to their practice by their local Clinical Research Network.

The GPWLS includes 14 questions related to job pressures, 10 questions related to job satisfaction, three questions related to intentions to quit direct patient care within the next five years, one question related to hours worked, and one question related to income from GP work (Table 1). These were included consistently across the four survey waves. The GPWLS also includes information on personal characteristics (gender, ethnicity, age, years from qualification) and employment status (partner versus salaried or locum).

**Table 1.** Measures of job pressures, job satisfaction, intention to quit, hours worked, income.

Outcome	Question	Domain
Job pressures	Please rate the following factors according to how much pressure you experience from each in your job^a^	Increased demands from patients Dealing with problem patients Dealing with earlier discharges from hospital Worrying about patient complaints/litigation Having insufficient time to do justice to the job Interruptions by emergency calls during surgery Unrealistically high expectation of role by others Insufficient resources within the practice Long working hours Paperwork Changes to meet requirements from external bodies (e.g. CQC, NHS England, CCG) Finding a locum Adverse publicity by the media Increasing workloads
Job satisfaction	Please indicate how satisfied you are with each of the following aspects of your job.^b^	Physical working conditions Freedom to choose your own method of working Your colleagues and fellow workers Recognition you get for good work Amount of responsibility you are given Your remuneration Opportunity to use your abilities Your hours of work Amount of variety in your job Taking everything into consideration, how do you feel about your job?
Intention to quit	What is the likelihood that you will make the following changes in your work life?^c^	Continue with medical work but outside the UK within five years? Leave direct patient care within five years? Leave medical work entirely within five years?
Hours worked	How many hours do you spend, on average, per week, doing NHS GP-related work? Please include ALL clinical and non-clinical NHS work	
Income	What is your total individual annual income from your job as a GP? This is the amount you receive before taxes but after deducting allowable expenses.^d^	

CQC: Care Quality Commission (the national regular); CCG: Clinical Commissioning Group (local commissioning organisation).

a 1–5, 1 = No pressure 2 = Slight pressure 3 = Moderate pressure 4 = Considerable pressure 5 = High pressure.

b 1–7, ranging from 1 = Extremely dissatisfied to 7 = Extremely satisfied.

c 1–5, 1 = None 2 = Slight 3 = Moderate 4 = Considerable 5 = High.

d 1 = less than £50,000, 2 = £50,000–£69,999, 3 = £70,000–£89,999, 4 = £90,000–£109,999, 5 = £110,000–£129,999, 6 = £130,000–£149,999, 7 = £150,000–£169,999, 8 = £170,000 or more.

Responses to individual questions were examined alongside three composite measures for job pressures, job satisfaction and intentions to quit direct patient care within the next five years. Job pressures and domains of job satisfaction were combined by taking the average score across all questions. Intention to quit was converted into a binary variable, with responses of considerable or high likelihood for any of the three questions coded as intentions to quit. Composite measures were coded as missing if there was a missing response for any individual question.

### Survey weighting

Responses to the GPWLS contain an over-representation of GP partners, and the 50–59-year-old age group (Supplementary Table 2). To account for this, we obtained the GP workforce headcount data from NHS England for the corresponding midpoint during the period when each survey was open to responses.^17^ We removed retired GPs and GP registrars, with the remaining GPs stratified by combinations of age, sex and contract type. We then used these data to generate survey weights to improve the representativeness of the sample.

### Deprivation ranking

We obtained data on deprivation for Lower-layer Super Output Areas (LSOAs) from the National Statistics English 2019 Indices of Deprivation.^18^ We used the income deprivation domain, representing the proportion of the population receiving welfare benefits due to low income. We used this measure of deprivation as this allowed calculation of the mean level of deprivation for a practice population. This is not feasible using overall Index of Multiple Deprivation scores as these are relative measures of deprivation.^19^

To attach area deprivation scores to practices, we obtained numbers of each practice’s registered patients that lived in each LSOA from NHS England.^20^ We then created a deprivation ranking based on average practice scores. The ranking has a uniform distribution between 0 and 1, where 0 is least deprived practice in England, 1 is most deprived, and 0.5 is the practice with the median deprivation score.

Using a deprivation rank for each practice offers several advantages over using practice deprivation scores. First, income deprivation scores can be skewed by outliers, where a few extreme values can disproportionately influence regression models. Ranking practices mitigates the impact of outliers, constraining the measure to a 0–1 scale and reducing the influence of extreme values. Second, the deprivation rank provides a relative measure of a practice's deprivation. For instance, a deprivation rank of 0.8 indicates that the practice is more deprived than 80% of other practices. This relative ranking enhances interpretability for policymakers. Third, they can be used to estimate Slope Indices of Inequality, which quantify the absolute differences in outcomes (i.e. job satisfaction, pressure levels, intention to quit, hours worked, or income reported) between the most and least deprived practices.^21,22^

### Analysis

Descriptive statistics for survey respondent characteristics were produced for each survey wave, using mean and standard deviations for characteristics with a normal distribution, and median and interquartile ranges for characteristics with a non-normal distribution. Analysis of Variance (ANOVA) tests were run to test for significant differences between survey waves. These analyses were also undertaken for practices grouped together into deprivation rank deciles.

We pooled data from all four waves and used linear regression to relate job pressures, job satisfaction, intentions to quit and hours worked to the deprivation rank of practices. Interval regression was used to relate reported income from GP work to the deprivation rank of practices as the outcome was measured in bands. Linear regression was performed using the STATA command ‘reg’, and interval regression was performed using STATA command ‘intreg’. Indicators were included for each survey wave within the regression models to account for differences in collection methods and events occurring during each survey wave including the COVID-19 pandemic during the fourth survey wave. We also included respondent characteristics (age, gender, ethnicity, employment status and years qualified) as covariates and used standard errors that were robust to heteroskedasticity. Gender (1 = female), ethnicity (1 = White) and employment status (1 = GP partner) were coded as binary variables. Age and years qualified were included using indicator variables for 10-year intervals. Probability weighting was used to incorporate survey weights within all models using the STATA subcommand ‘pweight’. In total, we ran 32 models to examine composite indicators and individual questions. We used the Bonferroni adjustment to adjust for multiple hypothesis testing and minimise the potential for type 1 error,^23^ and only considered *p* values of <0.0015625 rather than *p* < 0.05 as significant. All analyses were conducted using STATA v18, and the relevant code used for all analyses is contained in a publicly available repository (https://github.com/mikeuk2024/GPdeprivationanalysis).

We included four supplementary analyses to test the robustness of our results and further examine the relationship between deprivation ranking and GP job pressures, job satisfaction, intention to quit, hours worked and income earned. First, we grouped the deprivation ranks into deciles to look for non-linearity in the relationships. Second, we ran the models without survey weights. Third, we ran our analysis using robust standard errors clustered at the practice-level to account for repeated responses from GPs within certain practices. Fourth, we ran our analysis without data from the 2021 survey wave as responses may have been influenced by the COVID-19 pandemic and had more missing data than other survey waves.

### Missing data

Complete observations for predictor variables (deprivation rank, age, gender, employment status, ethnicity, years qualified) were present for 88% of responses (Supplementary Table 3). The 2021 survey wave had more missing data, with only 74.04% of responses having complete information for predictor variables. Among observations that had complete observations for predictor variables, less than 5% had missing observations for each outcome measure (Supplementary Table 4). We therefore adopted complete case analysis, but also ran our analysis without 2021 data as this survey wave was subject to more missing data than previous survey waves.

### Patient and public involvement

No patients were involved in the study design as it is focused on the perspectives of GPs. Groups of GPs working at the University of Manchester were consulted during the design of the GPWLS and piloted the survey prior to dissemination across the wider GP community.

## Results

### Descriptive statistics

There were only small differences in respondent characteristics across survey waves (Table 2). The exception was a lower proportion of GP partners responding in 2021 compared with later waves (69% in the 2021 wave compared with 81% across all waves). There were also only small differences in outcome measures between survey waves, except for high or considerable intention to quit over the next five years, which was lowest in 2021 (mean value of 37% in 2021 wave compared with 44% across all survey waves). The lower value in 2021 may be explained by the lower proportion of GP partner respondents in this wave as GP partners are typically older than salaried GPs and therefore closer to retirement age.

**Table 2.** Descriptive statistics of variables by survey wave.

Variable		Full sample		2015 survey		2017 survey		2019 survey		2021 survey		ANOVA test	
												*F*-stat	*p* > *F*
Deprivation rank^a^	Mean (SD)	0.44	(0.28)	0.48	(0.28)	0.44	(0.28)	0.40	(0.27)	0.42	(0.27)	29.07	0.000
Pressure composite score^b^	Mean (SD)	3.87	(0.69)	3.98	(0.63)	3.92	(0.66)	3.81	(0.73)	3.74	(0.73)	57.89	0.000
Satisfaction composite score^c^	Mean (SD)	4.73	(1.06)	4.64	(1.02)	4.72	(1.07)	4.85	(1.11)	4.78	(1.07)	12.86	0.000
Intention to quit composite score^d^ (% considerable or high intention)	Mean (SD)	43.52	(49.58)	45.54	(49.81)	46.17	(49.86)	45.54	(49.82)	37.42	(48.40)	15.74	0.000
Hours worked per week	Median (IQR)	40	(18)	43	(16)	40	(18)	40	(18)	40	(18)	27.04	0.000
Income from GP work^e^	Median (IQR)	4	(3)	4	(3)	4	(3)	4	(2)	3	(3)	14.84	0.000
Gender (% Female)	Mean (SD)	50.55	(50.00)	46.77	(49.91)	48.02	(49.97)	48.48	(50.00)	58.61	(49.26)	27.36	0.000
Ethnicity (% White)	Mean (SD)	81.52	(38.82)	82.49	(38.02)	81.95	(38.47)	82.84	(37.72)	79.19	(40.61)	3.88	0.009
Employment (% Partner)	Mean (SD)	80.82	(39.37)	87.04	(33.60)	86.98	(33.66)	77.70	(41.64)	69.44	(46.08)	111.05	0.000
Years qualified	Median (IQR)	22	(16)	24	(15)	23	(14)	24	(13)	18	(17)	74.89	0.000
Age (years)	Median (IQR)	50	(13)	51	(12)	51	(12)	52	(12)	47	(15)	87.91	0.000
Number of observations		8578		2615		2289		1390		2284			

GP: general practitioner; SD: standard deviation; IQR: interquartile range.

^a^Deprivation rank is a value between 0 and 1, where 0 is least deprived practice, 1 is most deprived, and 0.5 is the practice with the median deprivation score.

^b^1–5, ranging from 1 = No pressure to 5 = High pressure.

^c^1–7, ranging from 1 = Extremely dissatisfied to 7 = Extremely satisfied.

^d^1 = Considerable or high intention to quit.

^e^Reported income from GP work is a categorical variable with the following values 1 = less than £50,000, 2 = £50,000–£69,999, 3 = £70,000–£89,999, 4 = £90,000–£109,999, 5 = £110,000–£129,999, 6 = £130,000–£149,999, 7 = £150,000–£169,999, 8 = £170,000 or more.

When pooling data from all survey waves and comparing differences in responses from the most and least deprived practices (Supplementary Table 5), responses from practices within the 10% of most deprived practice populations nationally were from GPs who were, on average, less likely to be partners (77% vs. 82%), and less likely to be of White ethnicity (69% vs. 90%). There were only small raw differences in outcomes, except for intention to quit in the next five years (45% vs. 42%) and median income from GP work (3 vs. 4, 3 = £70,000–£89,999, 4 = £90,000–£109,999).

### Main results

**Figure 1.**
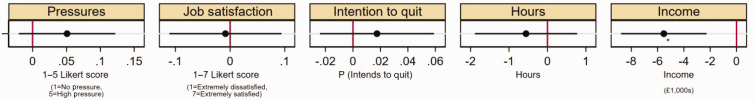
Co-efficient plot of deprivation rank against GP job pressures, job satisfaction, intentions to quit direct patient care, hours worked per week and reported income. Note: *p < 0.0015625, **p < 0.0003125, ***p < 0.00003125. Results are estimated using linear regression with robust standard errors except for income for which interval regression is used. Adjustment was made for individual characteristics including adjustment for Gender, Ethnicity, GP Partner status, Years since qualified, Age. The co-efficient presented is from the pooled analysis involving all survey waves. Full regression results are included in Supplementary Table 6.

When pooling all survey data together, there were no statistically significant associations between deprivation ranking and composite scores for job pressures (0.051, 95% CI: −0.020, 0.122), job satisfaction (−0.008, 95% CI: −0.111, 0.094), and intentions to quit (0.017, 95% CI: −0.024, 0.059) (Figure 1). There was also no statistically significant association between deprivation ranking and hours worked (−0.558, 95% CI: −1.887, 0.772), but there was a statistically significant negative association with reported GP income (−£5,525, 95% CI: −£8,773, −£2,276). Full regression results are provided in Supplementary Table 6.

**Figure 2.**
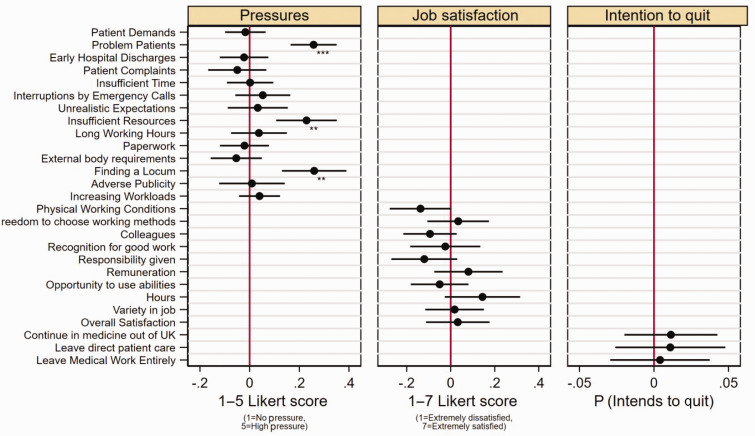
Co-efficient plot of deprivation rank against individual questions for GP job pressures, job satisfaction and intentions to quit direct patient care. Note: *p < 0.0015625, **p < 0.0003125, ***p < 0.00003125. Pressures scored from 1 to 5, ranging from 1 = No pressure to 5 = High pressure. Job satisfaction scored from 1 to 7, ranging from 1 = extremely dissatisfied to 7 = extremely satisfied. Results are estimated using linear regression with robust standard errors. Intention to quit variables are coded as a binary variable with 1 = if respondents answered considerable or high intention to quit. Adjustment was made for individual characteristics including adjustment for Gender, Ethnicity, GP Partner status, Years since qualified, Age. The co-efficient presented is from the pooled analysis involving all survey waves. Full regression results are included in Supplementary Tables 7–11.

When analysing the 14 specific sources of job pressure (Figure 2), deprivation ranking was associated with significantly higher pressures related to perceived problem patients (0.258, 95% CI: 0.165, 0.350), insufficient resources within the practice (0.229, 95% CI: 0.107, 0.351), and finding a locum (0.260, 95% CI: 0.130, 0.390). There were no statistically significant associations between deprivation ranking and the 10 domains of job satisfaction, or three forms of intention to quit direct patient care within the next five years. Full regression results are included in Supplementary Tables 7–11.

When comparing drivers of job pressures over time using the 2015 survey wave as a baseline, job pressures from perceived problem patients were similar between survey waves except for a small significant reduction in the 2019 survey wave (−0.089, 95% CI: −0.172, −0.006), pressures from insufficient resources in the practice were reduced in the 2019 (−0.130, 95% −0.235, −0.026), and 2021 survey waves (−0.266, 95% CI: −0.346, −0.186), and pressures from finding a locum initially increased in 2017 (0.497, 95% CI: 0.404, 0.590), and then reduced in the 2019 (−0.356, 95% CI: −0.478, −0.233) and 2021 survey waves (−0.620, 95% CI: −0.715, −0.525).

### Supplementary analyses

Re-estimating our models using deprivation deciles did not significantly change our findings (Supplementary Tables 11–16). GPs working in practices with the 10% most deprived populations reported statistically significant reduced income from GP work when compared with responses from the 10% least deprived practices (−£6,464, 95% CI: −£11,189, −£1,739). Focusing on individual questions, GPs serving the 10% most deprived populations reported statistically significant increased job pressures from perceived problem patients (0.266, 95% 0.135, 0.396), and insufficient resources within the practice (0.326, 95% 0.146, 0.506). Respondents from the 10% most deprived populations also reported increased job satisfaction with their hours of work (0.245, 95% 0.006, 0.484).

Re-estimating our models without survey weights (Supplementary Tables 17–23) also did not significantly change our findings, except for statistically significant associations of deprivation rank with reduced satisfaction with their colleagues and fellow workers (−0.110, 95% −0.212, −0.009) and reduced job pressures resulting from changes to meet requirements from external bodies (−0.090, 95% −0.162, −0.018). Using clustered standard errors at the practice level (Supplementary Tables 24–29) produced very similar results to our main analysis except for slightly wider confidence intervals, suggesting that our analysis was not significantly impacted by repeated respondents from the same practices. Excluding responses to the 2021 survey wave from our analysis (Supplementary Tables 30–35) produced similar results, except for the association between deprivation ranking and reported GP income (−£5,248, 95% CI: −£9,007, −£1,489). This was no longer considered statistically significant as the *p*-value was not less than 0.0015625.

## Discussion

### Summary of findings

Our findings demonstrate that population deprivation is significantly associated with certain job pressures on GPs, including dealing with perceived problem patients, insufficient resources in the practice and finding a locum. Population deprivation was also associated with reduced income from GP work. There were no significant associations between population deprivation and other sources of job pressures, factors influencing job satisfaction, intentions to quit direct patient care, or hours worked.

### Strengths and limitations

To our knowledge, this is the first quantitative analysis examining the relationship between population deprivation and the working lives of GPs in England. However, there are some limitations of this analysis, which need to be acknowledged. First, respondents to the GPWLS may not be representative of the full GP population in England. We attempted to mitigate this issue using survey weights. Second, there is potential for selection bias within the GPWLS, as it is possible that GPs experiencing greater job pressures may have less time to engage with the survey or GPs with lower job satisfaction may be more likely to engage with the survey as the GPWLS is framed as an opportunity to encourage policy change. Third, there are differences between the survey waves used in this analysis. Pressure was measured at different times of the year for each survey, and the collection method moved to an online survey in the 2021 survey. The 2021 survey was also impacted by the COVID-19 pandemic, which placed GPs under considerable pressure as they were faced with rapidly redesigning services to limit transmission of COVID-19. The overall provision of appointments in general practice increased during the pandemic, although differences in consultation rates between the most and least populations narrowed.^24^ Most deprived populations also saw a relatively greater increase in remote consultation rates compared with the least deprived populations.^24^ Qualitative research has also provided important insights into mechanisms through which socioeconomic deprivation exposed patients in general practice to an increased risk of COVID-19, including greater prevalence of underlying health conditions such as smoking-related respiratory diseases, geographical barriers to accessing COVID-19 assessment centres, and poor language skills and digital literacy, meaning remote and online access to primary care services was problematic.^25^ We took account of differences between survey waves by including indicators for each survey wave. We also ran our models with data from the 2021 survey wave to ascertain if this changed our results. Finally, while we provide useful insights based upon responses to individual questions, some questions are broad and may be interpreted in different ways by alternative GPs. For example, the GPWLS does not define problem patients nor allow respondents to detail which specific resource constraints may drive GP pressures.

### Comparison to pre-existing literature

There have only been a few previous studies that have examined working conditions of GPs in areas of greater deprivation.^15,16^ The most prominent example remains qualitative work in Scotland interviewing GPs at the ‘Deep End’, comprising GPs serving the 100 most deprived practice populations in Scotland.^15^ A consistent theme throughout this research has been GPs reporting a mismatch between resources and needs when working in areas of deprivation, a common finding with our study. There is also literature to support our significant finding related to working in areas of greater deprivation and job pressures related to struggling to find a locum. Nussbaum et al. found fewer GPs per 1000 population in areas of greater deprivation,^10^ and Parisi et al. found higher GP turnover in deprived areas.^11^ Recruitment and retention of GPs working in areas of greater deprivation has also been a challenge in Scotland. While these studies do not focus specifically on GP locums, they indicate a general reduced supply of GPs working in areas of greater deprivation, which likely corresponds to greater demand for GP locum staff.

Previous analysis of GP incomes between 2011 and 2013 found only small differences by deprivation.^26^ Our more recent analysis provides compelling evidence of income disparities between GPs working in areas of greater and lesser deprivation of practice population. This is important as reduced income may be one factor driving some of the GP recruitment and retention challenges faced by practices in areas of greater deprivation.

### Implications for research and practice

Our finding related to perceived problem patients requires further research to investigate the exact drivers of individuals being labelled as problem patients, implications for care, and strategies to address this. The concept of problem patients has historically has been defined as patients whose behaviours deviate from ‘expectations which are shared and recognised as legitimate within the social system’.^27^ Although GPs labelling individuals as problem patients may be unhelpful in practice, the term may be better understood as resulting from misalignment of expectations, behaviours and beliefs between patients and GPs or a failure of the healthcare system to meet the needs and expectations of patients with complex lives and needs.

The question related to intentions to quit in the GPWLS asks about intentions to quit direct patient care entirely or the UK over the next five years, and therefore do not capture intentions related to changing employment away from areas of greater deprivation. More research is needed to understand the specific drivers of higher turnover of GPs in areas of greater deprivation to inform policy development that aims to improve recruitment and retention. This could be achieved through exit interviews and surveys, and mandatory data collection processes from these processes.

General practice in England is facing a GP workforce crisis, with declining numbers of GPs and rising patient demand.^28^ The GP workforce crisis is known to be worse in practices within areas of greater deprivation, which have fewer GPs per capita and greater GP turnover. If not addressed, the disparity in GP recruitment and retention between areas of greater and lesser deprivation will likely contribute to worsening inequalities in the supply of high-quality healthcare services described by the inverse care law. There are several potential solutions that could be considered to address these disparities and promote the equitable supply of healthcare staff within general practice and the NHS more generally. As we find that GPs working in areas of greater deprivation report lower earnings and experience greater job pressures related to insufficient resources, policymakers may consider including deprivation as a needs-based factor within general practice resource allocation formula. Deprivation is already included within the resource allocation formula used for Integrated Care Boards,^29^ and doing the same for general practice may improve funding and promote more equitable supply of GP staff in areas of greater deprivation compared with areas of lesser deprivation. However, financial incentives need to be combined with other measures such as undergraduate and postgraduate training opportunities in areas of greater deprivation, implementing appropriate professional and personal support, and protected time for continuing professional development. Alongside acknowledging challenges, there also needs to be broader recognition by the GP community of potential advantages of working in areas of greater deprivation such as the rewarding nature of the work, positive relationships with patients, and greater confidence and skills from managing complex clinical scenarios.^30^

## Declarations

### Guarantor

MA.

### Data availability statement

Data from the GPWLS are not publicly available. Information on the GP workforce and practice populations are publicly available on the NHS England website.

### Provenance

Not commissioned; peer-reviewed by Julie Morris and Anant R Jani.
